# Facilitating Water Permeation in Graphene Oxide Membranes via Incorporating Sulfonato Calix[n]arenes

**DOI:** 10.3390/membranes14020032

**Published:** 2024-01-24

**Authors:** Yufan Ji, Shurui Dong, Yiping Huang, Changhai Yue, Hao Zhu, Dan Wu, Jing Zhao

**Affiliations:** 1 China Construction Industrial & Energy Engineering Group, Nanjing 210023, China; jyfqsa@163.com (Y.J.); ych1987627@163.com (C.Y.); zhu-hao@cscec.com (H.Z.); wudan8587@163.com (D.W.); 2 State Key Laboratory of Materials-Oriented Chemical Engineering, College of Chemical Engineering, Nanjing Tech University, Nanjing 211816, China; srd@njtech.edu.cn

**Keywords:** graphene oxide, membrane, sulfonato calix[n]arenes, pervaporation, water channel

## Abstract

Graphene oxide (GO) with its atomic thickness and abundant functional groups holds great potential in molecular-scale membrane separation. However, constructing high-speed and highly selective water transport channels within GO membranes remains a key challenge. Herein, sulfonato calix[n]arenes (SCn) molecules with a cavity structure, hydrophilic entrance, and hydrophobic wall were incorporated into GO interlayer channels through a layer-by-layer assembly approach to facilitate water permeation in a water/ethanol separation process. The hydrophilic entrance enables preferential access of water molecules to the cavity over ethanol molecules, while the high hydrophobicity of the cavity wall confers low resistance for water diffusion. After incorporating SCn molecules, the membrane shows a remarkable increase in the water/ethanol separation factor from 732 to 1260, while the permeate flux also increases by about 50%. In addition, the strong electrostatic interactions between the building blocks endow the membrane with excellent swelling resistance even under a high water content. This work provides an effective strategy of constructing high-efficiency water transport channels in membrane.

## 1. Introduction

Ethanol produced from biomass is a promising renewable fuel which can reduce the consumption of crude oil and alleviate environmental pollution [1]. A dehydration process is needed before the produced ethanol can be used as fuel [2]. Due to the formation of water/ethanol azeotropes at an ethanol concentration of 89.4 mol% (95.6 wt%, at 1 atm and 78.2 °C), special distillation technologies such as azeotropic distillation and vacuum distillation have been employed to obtain high-purity ethanol, which are highly energy-intensive [3,4]. Comparatively, pervaporation membrane technology is an attractive approach for solvent dehydration and purification due to its low energy consumption, small footprint, environmental friendliness, and high separation efficiency [5,6,7].

In recent years, the exploration of advanced materials has boosted the growing development of high-performance membranes [8]. For instance, two-dimensional (2D) materials represented by graphene oxide (GO) have been widely used as building blocks to construct ordered and tailorable channels for the fast transport of water/gas molecules and ions [9,10,11]. The engineering of water transport channels in the GO membrane is critical to the realization of high-efficiency water/alcohol separation. In this regard, different kinds of molecules (such as cationic polyelectrolytes and small molecules with reactive groups) have been incorporated into the interlayer channels of the GO membrane. The interlayer channel size can be precisely tuned to improve the molecular sieving ability, while the chemical environment of the channel can be optimized to provide high water affinity and thus leading to enhanced adsorption selectivity. The synergistic optimization of these two factors could obtain superior separation performance, especially in the separation factor [12,13,14]. The high aspect ratio of GO nanosheets leads to tortuous mass transfer channels in GO-based membranes, which partially offset the advantage of an ultrathin separation layer in improving permeate flux [15]. To address this issue, various strategies have been adopted to decrease diffusion resistance and achieve a high flux. Huang et al. [16] introduced a highly hydrophilic spherical polyelectrolyte brush (SPB) into GO interlayer channels. The excellent hydrophilicity of SPB nanospheres promotes water sorption, while the flexible polymer chains enlarge the channels and facilitate water diffusion, resulting in the simultaneous enhancement of flux and selectivity. In our previous work [17], we proposed a facile approach of co-assembling GO and porous GO nanosheets to increase the number of defects and slits, which provide more pathways for molecular transport and lead to a doubling of the permeate flux.

**Figure 1:**
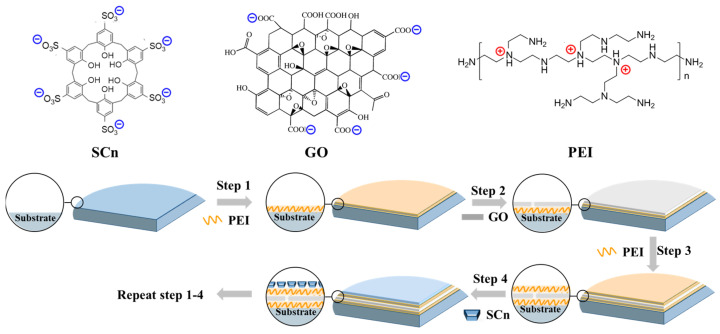
Molecular structure of building blocks and schematic illustration of the membrane fabrication process (only the partial membrane structure adjoining substrate was displayed in the figure).

Calix[n]arene is a type of organic macrocyclic compound with sub-nanometer or nanometer scale cavities. The cavities are constructed by multiple benzene rings, which show high hydrophobicity and confer low resistance for water diffusion [18,19]. After being functionalized with sulfonate groups, calix[n]arene molecules (sulfonato calix[n]arenes, SCn) possess excellent water solubility and the entrance to the cavity turns to be highly hydrophilic [20,21], thus promoting the selective transport of water molecules. Therefore, the combination of a hydrophilic entrance and a hydrophobic wall enables the SCn molecules to be ideal water transport channels [22,23]. Lan et al. [23] found that the water flux of a thin-film composite (TFC) membrane was significantly increased after incorporating SCn molecules. In this work, to facilitate water permeation through a GO membrane in the water/ethanol separation process, we introduced SC6 molecules into GO interlayer channels with the assistance of cationic polyelectrolyte polyethyleneimine (PEI) (Figure 1). The strong electrostatic interactions between GO/PEI and SC6/PEI ensure a stable membrane structure, while the existence of SC6 molecules provide abundant channels for the preferential transport of water molecules, thus leading to a remarkable improvement in both the permeate flux and water/ethanol separation factor.

## 2. Materials and Methods

### 2.1. Materials

GO was purchased from Nanjing Jicang Nano Technology Co., Ltd., Nanjing, China. SCn (*n* = 4, 6, and 8) were bought from TCI (Shanghai) Huacheng Industry Development Co., Ltd., Shanghai, China. PEI molecules with different molecular weights (Mw = 1800, 10 k, and 70 kDa) were provided by Alfa Aesar (Shanghai, China) Chemical Co., Ltd., Shanghai, China. Sodium hydroxide (NaOH) was obtained from Sinopharm Chemical Reagent Co., Ltd., Shanghai, China. Deionized water used in the laboratory was homemade. The flat-sheet polyacrylonitrile (PAN) ultrafiltration membrane with a molecular weight cutoff of 100 kDa was received from Shandong Mega Vision Membrane Engineering & Technology Co., Ltd., Taian, China.

### 2.2. Preparation of GO, PEI/GO, and PEI/GO/SC6 Membranes

**Figure 2:**
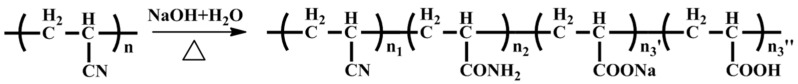
The reaction mechanism of PAN hydrolysis.

Both the SCn molecules and GO nanosheets are negatively charged, so the cationic polyelectrolyte PEI was used to assist their deposition on the PAN substrate through a layer-by-layer self-assembly method. Firstly, the hydrolysis of PAN substrate was carried out via immersing PAN in 1.5 M NaOH solution at 328 K for 30 min to partially convert cyano groups to carboxyl groups (Figure 2) [24,25]. Meanwhile, GO, PEI, and SC6 aqueous dispersions/solutions with the concentration of 0.2 mg/mL were prepared. Specifically, the GO dispersion was prepared via adding GO powder into water, followed by ultrasonication (400 W, 30 min) and centrifugation (3500 rpm, 10 min) to remove aggregated GO flakes. The assembly process was performed in the sequence of PEI–GO–PEI–SC6 via spin coating (300 rpm, 9 s; 2000 rpm, 60 s). After 5 cycles of assembly, a dense and defect-free separation layer was formed on the substrate. Deionized water was used after each spin-coating step to remove the molecules/nanosheets physically attached to the surface. Finally, the prepared membranes were vacuum-dried and stored before test. The membranes were named as PEI/GO/SC6. Meanwhile, PEI and GO were alternately spin-coated for 5 and 10 cycles to prepare PEI/GO(5) and PEI/GO(10) membranes as control membranes.

### 2.3. Characterization

The surface and cross-section morphologies of the prepared membranes were characterized by using field emission scanning electron microscopy (FESEM, Hitachi Limited, S-4800, Tokyo, Japan). The membrane thickness was determined by measuring at least ten different locations in the cross-sectional SEM images and taking an average value. Atomic force microscopy (AFM, Park SYSTEMS, Suwon, Republic of Korea) was used to obtain the height profiles of GO nanosheet. The AFM samples were prepared via diluting the GO dispersion for membrane fabrication to an ultralow concentration (1 × 10^−5^ mg/mL), depositing the GO dispersion on a silicon wafer and then drying it at room temperature. The chemical compositions of the PEI/GO/SCn membranes were analyzed by using X-ray photoelectron spectroscopy (XPS, Thermo ESCALAB 250, Waltham, MA, USA) and Fourier transform infrared (FTIR, AVATAR-FT-IR-360, Thermo Nicolet, Waltham, MA, USA).

### 2.4. Pervaporation Experiment

The membrane performance was investigated by using pervaporation separation of the ethanol/water mixture. The membrane with an effective area of 2.54 × 10^−4^ m^2^ was placed in a homemade membrane module. Then, the solution with 10 wt% water was fed continuously to the membrane module by using peristaltic pump. The permeate side of the membrane was evacuated with a pressure of less than 300 Pa to provide driving force for the molecular permeation across membrane. When the membrane reached a steady state, a cold trap was used to collect the vapor on the permeate side, which was then weighed by using a balance and analyzed by using gas chromatography (SP-6890, Shandong Lunan Analytical Instrument Co., Ltd., Tengzhou, China). The evaluation parameters for membrane performance (permeate flux *J* (kg/m^2^h) and separation factor *α*) were obtained by the following equations:

(1) $$J=\frac{M}{A\times t}$$

(2) $$\alpha =\frac{y_{W}/y_{A}}{x_{W}/x_{A}}$$

where *M* (kg) is the mass of collected permeate, *A* (m^2^) is the effective area of the membrane, and *t* (h) is the operation time. *y_W_* is the mass fraction of water in the permeate; *x_W_* is the mass fraction of water in the feed; and *y_A_* and *x_A_* represent the mass fractions of ethanol in the permeate and feed, respectively. To guarantee the validity of experimental data, the pervaporation experiments were repeated with three parallel samples.

The permeance (driving force-normalized form of permeation flux) of individual components ((*P/l*)*_i_*, GPU) (1 GPU = 7.501 × 10^−12^ m^3^(STP)/(m^2^·s·Pa)) and selectivity (*β*) were defined as follows:

(3) $${\left(P/l\right)}_{i}=\frac{J_{i}}{p_{i0}-p_{il}}=\frac{J_{i}}{\gamma_{i0}x_{i0}p_{i0}^{sat}-p_{il}}$$

(4) $$\beta =\frac{{\left(P/l\right)}_{W}}{{\left(P/l\right)}_{E}}$$

where *J_i_* (g/(m^2^·h)) is the permeate flux of component *i*, *l* (m) is the thickness of membrane, *p*_*i*0_, *p_il_* (Pa) are the partial pressures of component *i* in the feed side and permeate side, and *p_il_* can be calculated approximately as 0 for the high vacuum degree in the permeate side. *γ*_*i*0_ is the activity coefficient of component *i* in the feed liquid that was obtained using Aspen Plus (Aspen Plus v11, Aspen Technology Inc., Bedford, MA, USA), and NRTL equation was used during calculation; *x*_*i*0_ is the mole fraction of the component *i* in the feed liquid; and $p_{i0}^{sat}$(Pa) is the saturated vapor pressure of pure component *i* at the operation temperature, which can be calculated through Antoine equation. The permeate flux of water and ethanol should be transformed into the volumes under standard temperature and pressure (STP): 1 kg of water vapor at STP = 1.245 m^3^ (STP) and 1 kg of ethanol vapor at STP = 0.487 m^3^ (STP).

## 3. Results and Discussion

### 3.1. Morphologies of GO Nanosheet and GO-Based Membranes

**Figure 3:**
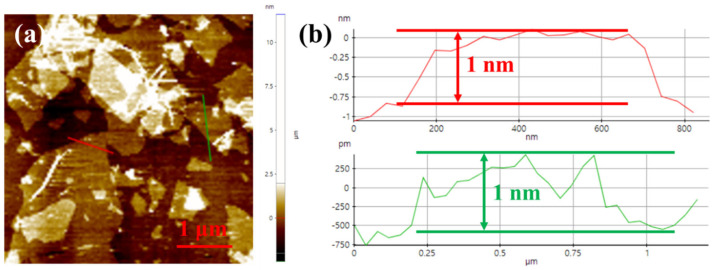
(**a**) AFM image of GO nanosheet; (**b**) height profiles of GO nanosheet.

The morphology of graphene oxide nanosheets after ultrasonic treatment was characterized by using AFM. As shown in Figure 3, the lateral size of the nanosheet is around 0.5–2 μm, and the thickness is about 1 nm. The high aspect ratio and the well-exfoliated structure of the GO nanosheet are conducive to its ordered assembly into ultrathin and defect-free membranes.

**Figure 4:**
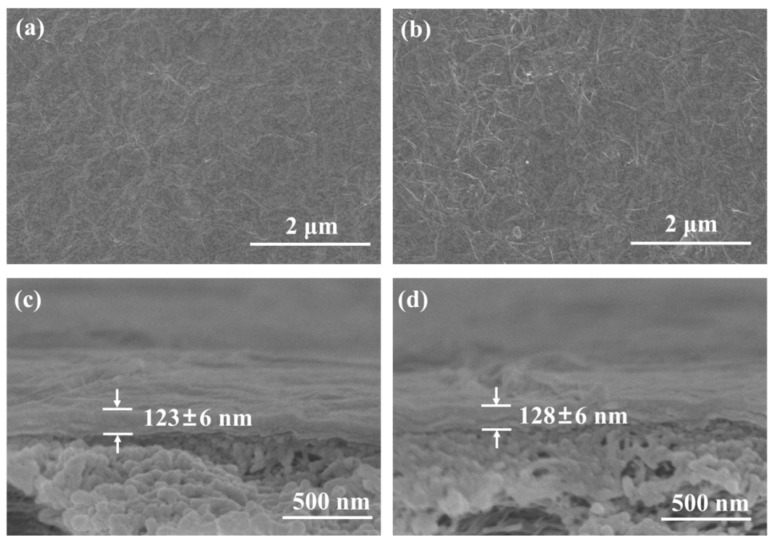
Surface SEM images of (**a**) PEI/GO(10) and (**b**) PEI/GO/SC6 membranes; Cross-sectional SEM images of (**c**) PEI/GO(10) and (**d**) PEI/GO/SC6 membranes.

As shown in Figure 4a,b, both the PEI/GO and PEI/GO/SCn membranes display a dense and defect-free surface morphology with abundant wrinkles, which are commonly observed in GO-based membranes and confirm the high ratio of GO in the membranes. The selective layer thicknesses of all the membranes are in the range of 110–130 nm (Figure 4c,d). Although the deposition amount varies with the change in building blocks, the tiny change in membrane thickness could not be accurately characterized by using SEM.

### 3.2. Chemical Compositions of GO-Based Membranes

**Figure 5:**
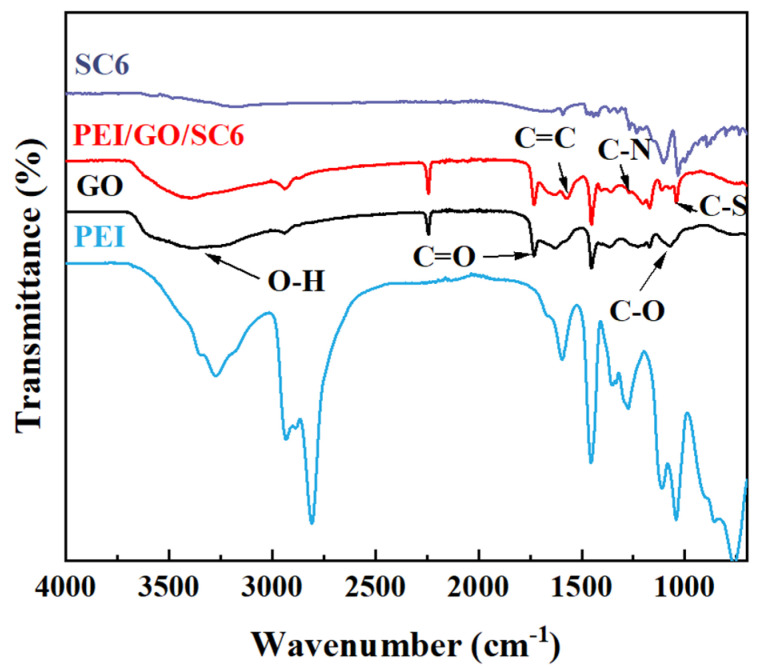
FTIR spectra of GO, PEI, SC6, and PEI/GO/SC6 membranes.

The characteristic peaks corresponding to the hydroxyl (–OH), carboxyl (C=O), and epoxy (C–O–C) groups were observed on the FT-IR spectrum of the GO membrane at the wavenumbers of 3365 cm^−1^, 1731 cm^−1^, and 1063 cm^−1^, respectively (Figure 5). By comparing the spectra of the GO and PEI/GO/SC6 membranes, it can be observed that some new peaks appear at the positions of 1567 cm^−1^, 1285 cm^−1^, and 1043 cm^−1^ after incorporating PEI and SCn. Among them, the absorption peaks at 1567 and 1043 cm^−1^ belong to the C=C and C–S bonds in the SC6 molecules, respectively, and the peak at 1285 cm^−1^ can be attributed to the C–N in PEI molecules. From the above analysis, it could be deduced that PEI, GO, and SCn were successfully assembled on the surface of the PAN substrate.

**Figure 6:**
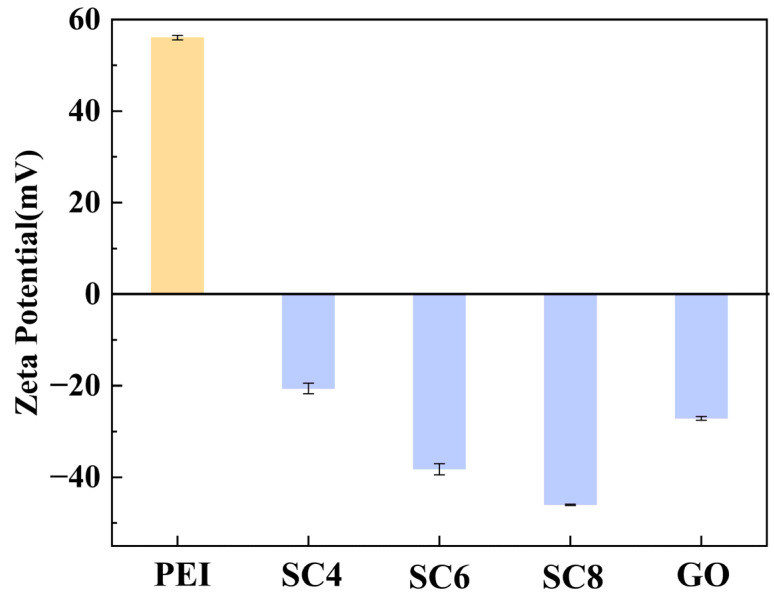
Zeta potential values of different membrane building blocks.

**Figure 7:**
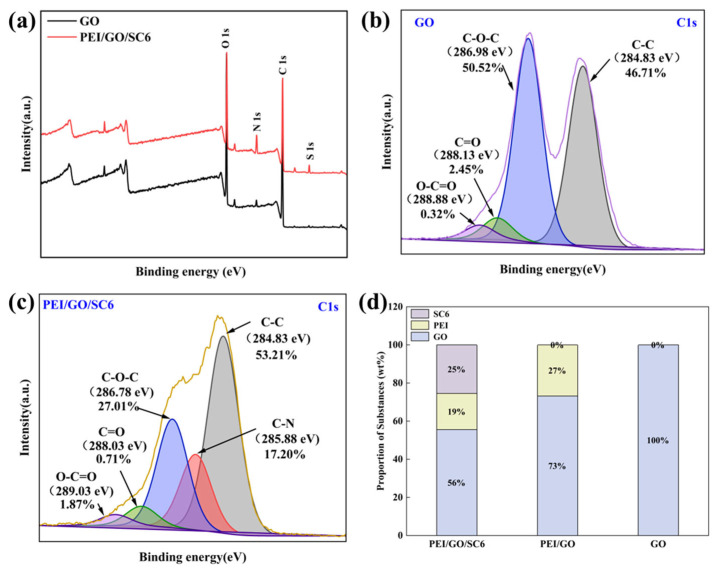
(**a**) XPS spectra of GO and PEI/GO/SC6 membranes; (**b**) C1s XPS spectrum of GO membrane; (**c**) C1s XPS spectrum of PEI/GO/SC6 membrane; (**d**) Proportions of different building blocks in membranes.

The zeta potential values of different building blocks (PEI, GO, and SCn) were measured as shown in Figure 6. PEI possesses abundant positive charges with a high zeta potential of 56 mV, while GO and SCn are negatively charged. Therefore, electrostatic interactions will be formed between PEI/GO and PEI/SCn, and PEI is an ideal candidate to be used as a binder between GO and SCn. The zeta potential of SCn molecules increases with the increasing amount of the phenol unit because each phenol unit is functionalized with one sulfonate group. The cavity sizes of SC4, SC6, and SC8 molecules are 3.0 Å, 7.6 Å, and 11.7 Å, respectively [23]. A moderate cavity size is desirable to provide low diffusion resistance while meanwhile conferring a certain sieving capacity. From the aspect of molecular structure, the four sulfonate groups of SC4 are located on the same side of the cavity, while for SC6 and SC8, the sulfonate groups are distributed on both sides of the cavity, which makes them more suitable to be used in the layer-by-layer assembly process [26,27]. Comprehensively considering the channel size, charge property, and molecular conformation, SC6 was employed in the following experiments:

The chemical compositions of membranes were intensively analyzed via XPS characterization (Figure 7). Compared with the spectrum of GO, the characteristic peaks of the N and S elements belonging to the PEI and SC6 molecules appear in the spectrum of the PEI/GO/SC6 membrane, which prove the existence of PEI and SC6 in the membrane. For an in-depth analysis, the C1s spectra of the GO and PEI/GO/SC6 membranes were obtained. The peaks at the binding energies of 284.83 eV, 286.98 eV, 288.13 eV, and 288.88 eV belong to the C–C, C–O–C, C=O, and O–C=O groups on GO, respectively, and the epoxy group almost accounts for half of the total groups (Figure 7b). With the introduction of PEI and SC6, a new C–N characteristic peak appears at the binding energy of 285.88 eV. In addition, the proportion of C–O–C shows a more remarkable decrease (from 51% to 27%) compared with other groups, which indicates that partial epoxy groups were consumed via reacting with the amino groups on PEI. Therefore, both the electrostatic interaction and covalent bond can be formed between PEI and GO. As for PEI/SC6, the amino groups on PEI are hard to react with the hydroxyl and sulfonate groups on SC6 at ambient temperature, so the driving force for the assembly of SC6 is electrostatic interaction. According to the contents of the elements S and N, the proportions of SC6, PEI, and GO in different membranes were calculated. As shown in Figure 7d, the mass of GO accounts for more than half in the PEI/GO and PEI/GO/SC6 membranes, revealing that GO is still the dominant component of the composite membrane. PEI accounts for the least among the three components, which implies that PEI mainly functions as a binder between GO nanosheets and SC6 molecules.

### 3.3. Pervaporation Performance of Membranes

#### 3.3.1. Effect of Substrate

In the membrane preparation process, positively charged PEI was deposited on the surface of the PAN substrate as the first layer. The deposition amount of PEI molecules and their coverage ratio on the PAN substrate are of key importance for the assembly of subsequent layers and thus influence the final membrane structure. A stronger interaction between PEI and the substrate can lead to a larger PEI deposition amount. In this light, the PAN substrate was treated with NaOH to convert PAN into hydrolyzed PAN (hPAN) and introduce abundant carboxyl groups on the surface, which can form strong electrostatic interactions with PEI molecules. As shown in Table 1, the PEI/GO/SC6 membrane with the hPAN substrate shows a remarkably improved separation performance with a permeate flux of 3.15 kg/(m^2^·h) and a water/ethanol separation factor of 1260. The permeate flux is 57% higher than that of the membrane with the pristine PAN substrate, while the separation factor is 3.8-times higher. For the membrane with the un-hydrolyzed PAN substrate, the low coverage ratio of the PEI layer leads to the inadequate deposition of GO and SCn, and meanwhile the small SCn molecules are prone to penetrating into the uncovered pores on the PAN surface. Even though a defect-free membrane structure can be formed after multiple deposition cycles, the mass transfer channels in the membrane are disordered, thus causing the much lower permeate flux and separation factor.

The molecular weight of PEI is 10,000 Da; the feed solution is water/ethanol (10/90 wt%) mixture; and the operation temperature is 343 K.

#### 3.3.2. Effect of Incorporating SC6 Molecules

**Figure 8:**
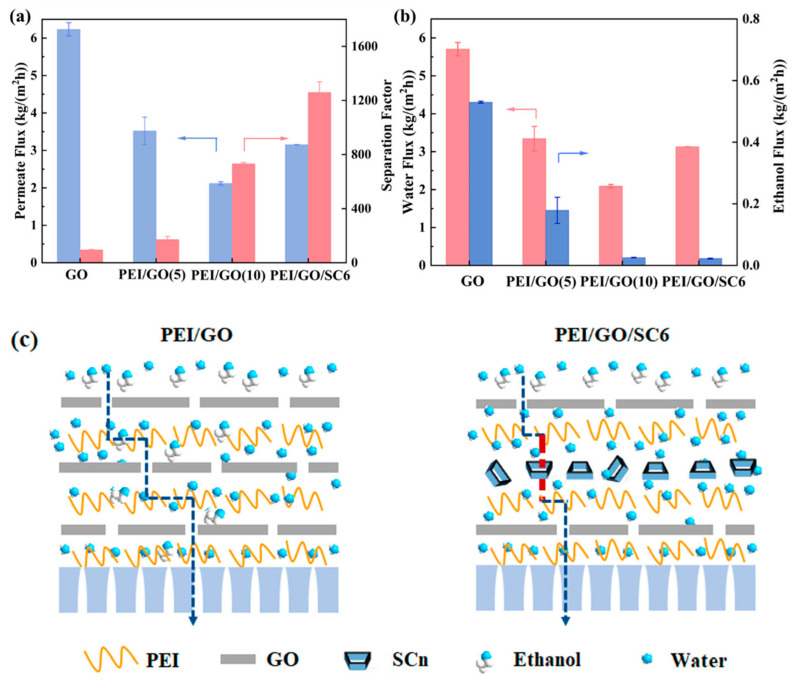
(**a**,**b**) The effect of SCn molecules on the separation performance of GO-based membranes. Feed water content: 10 wt% and feed temperature: 343 K. Red and blue arrows illustrate which parameter has been measured and shown on the figure. (**c**) Schematic diagram of molecular transport in PEI/GO and PEI/GO/SC6 membranes (only the partial membrane structure adjoining substrate was displayed in the figure).

The PEI/GO/SC6 membrane was prepared to investigate the influences of SCn molecules on membrane separation performance. In addition, the pristine GO membrane and the PEI/GO membranes without SCn were prepared for comparison via suction filtration and layer-by-layer self-assembly methods, respectively. As shown in Figure 8a, the water/ethanol separation factor of the GO membrane is as low as 97 with the flux up to 6.23 kg/(m^2^·h), which can be ascribed to the weak interlayer bonding and the resultant swollen membrane structure during operation. After incorporating PEI into the GO interlayer channels, the electrostatic interactions and covalent bonds between PEI and GO could play a role in stabilizing membrane structure to achieve efficient water/ethanol separation. In addition, the excellent hydrophilicity of PEI could promote the preferential adsorption of water molecules in the membrane. As a result, the PEI/GO(10) membrane shows a water/ethanol separation factor of 732, which is 6.5 times higher than that of the pristine GO membrane. To more clearly demonstrate the role of SCn molecules, we prepared a PEI/GO/SC6 membrane with five cycles of assembly in the order of PEI–GO–PEI–SC6 to compare with the PEI/GO(10) membrane. In both membranes, PEI assembly was repeated for 10 times. The difference is that there are 10 GO layers in PEI/GO(10), while there are 5 GO layers and 5 SC6 layers in the PEI/GO/SC6 membrane. Compared with the PEI/GO(10) membrane, the PEI/GO/SC6 membrane shows a remarkable increase in separation factor from 732 to 1260, while the permeate flux also increases by about 50%, confirming the critical role of SC6 molecules in facilitating water permeation. The significant improvement in separation performance can be ascribed to two reasons: (1) The GO nanosheets with a large aspect ratio in the PEI/GO membrane construct tortuous mass transfer channels, which is alleviated with the incorporation of SC6, thus decreasing the diffusion resistance. (2) The abundant sulfonate groups around the cavity opening of SC6 provide high water affinity and ethanol repulsion, while the cavity wall is hydrophobic due to the existence of benzene rings. The combination of a hydrophilic opening and a hydrophobic wall enables the easy access of water molecules to the cavity and fast diffusion through it. As a result, the water flux remarkably increases, while ethanol flux decreases, leading to the simultaneous enhancement of the permeate flux and separation factor. In addition, the strong electrostatic interaction among SC6, PEI, and GO ensures the structural stability of the mass transfer channels during operation. The PEI/GO(5) membrane was also prepared to compare with the PEI/GO/SC6 membrane because there are five GO layers in both membranes. The deposition amount during each cycle of the PEI/GO assembly is small, so multiple cycles are needed to completely cover the pores on the substrate surface and then form a defect-free separation layer. With five cycles of assembly, there are still some defects in the membrane, leading to the poor separation factor of 172. Compared with the PEI/GO(5) membrane, the PEI/GO/SC6 membrane shows a 6.3-times higher separation factor, while the permeate flux only decreases by 10%.

#### 3.3.3. Effect of PEI Molecular Weight

**Figure 9:**
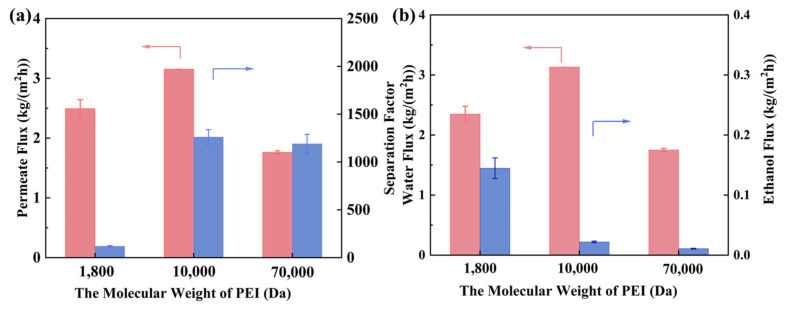
Effect of the PEI molecular weight on the separation performance of PEI/GO/SC6 membranes: (**a**) permeate flux and separation factor, (**b**) water flux and ethanol flux. Feed water content: 10 wt%, feed temperature: 343 K. Red and blue arrows illustrate which parameter has been measured and shown on the figure.

During the assembly of the PEI/GO/SCn membranes, PEI is a critical component because it interacts with both GO and SCn and determines the final membrane structure. The effect of PEI molecular weight on the separation performance of the PEI/GO/SC6 membrane was investigated. As shown in Figure 9, only when the PEI molecular weight is higher than 10 kDa can the membrane achieve efficient water/ethanol separation with the separation factor reaching 1200, while the separation factor is only 119 when employing 1800 Da PEI molecules. The possible reason is that PEI molecules with a higher molecular weight possess more interaction sites and longer molecular chains. They are more easily adsorbed on the hPAN surface and then achieve a higher coverage ratio, which is in favor of the subsequent assembly of GO and SCn. Consequently, an ordered and a defect-free membrane structure can be formed, which inhibits the diffusion of ethanol molecules (Figure 9b). When the PEI molecular weight increases to 70 kDa, the separation factor almost keeps constant, yet the denser membrane structure increases the diffusion resistance and leads to the remarkable decline in permeate flux. Therefore, the membrane with a PEI molecular weight of 10 kDa obtains the optimal separation performance with the permeate flux of 3.15 kg/(m^2^·h) and separation factor of 1260.

#### 3.3.4. Effect of Operation Temperature and Water Content in Feed

**Figure 10:**
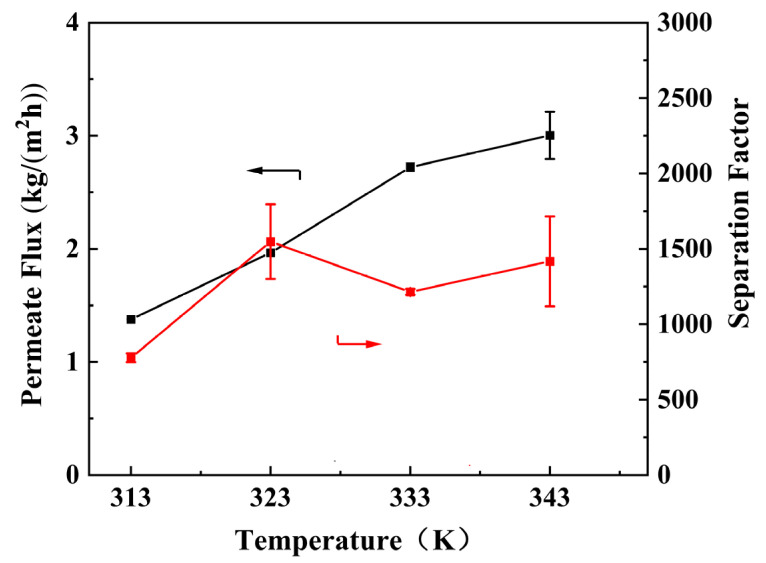
Effect of operation temperature on the separation performance of PEI/GO/SC6 membrane. Red and black arrows illustrate which parameter has been measured and shown on the figure.

The separation performance of the PEI/GO/SC6 membrane at different operation temperatures was evaluated with the water content in the feed of 10 wt% (Figure 10). The permeate flux remarkably increases with increasing temperature, while the water/ethanol separation factor increases firstly and then fluctuates in a narrow range. In order to deeply understand the molecular permeation behavior at different temperatures, we calculated the permeability (driving force-normalized form of permeate flux) and selectivity values of the membrane. As shown in Table 2, both water permeability and ethanol permeability continuously decline with increasing temperature. The molecular permeation across the membrane can be divided into two steps: adsorption and diffusion, while the increasing temperature exerts reverse impacts on these two processes: higher temperature inhibits molecular adsorption and meanwhile favors molecular diffusion. The declined permeability with increasing temperature demonstrates that the negative impacts on adsorption play a key role. In addition, this result also confirms the stable membrane structure at a higher temperature, because the obvious swelling of the membrane structure will lead to remarkably facilitated molecular diffusion and elevated permeability.

**Figure 11:**
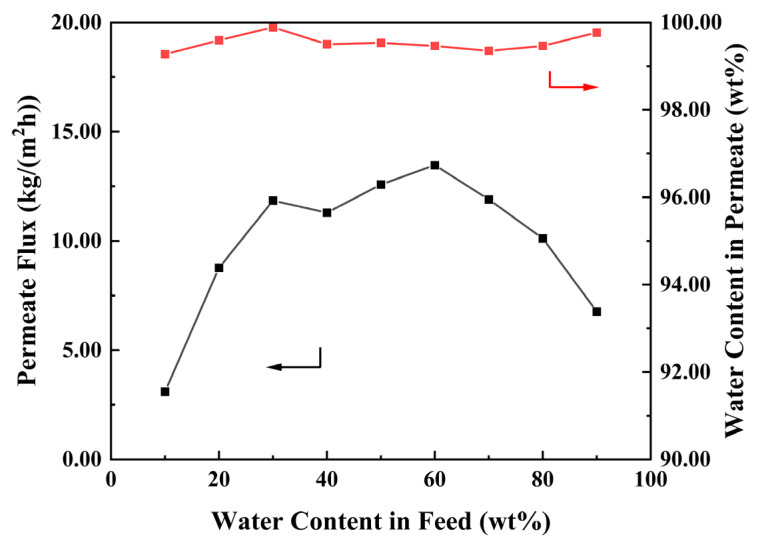
Effect of the water content in feed on the separation performance of PEI/GO/SC6 membrane. Red and black arrows illustrate which parameter has been measured and shown on the figure.

**Figure 12:**
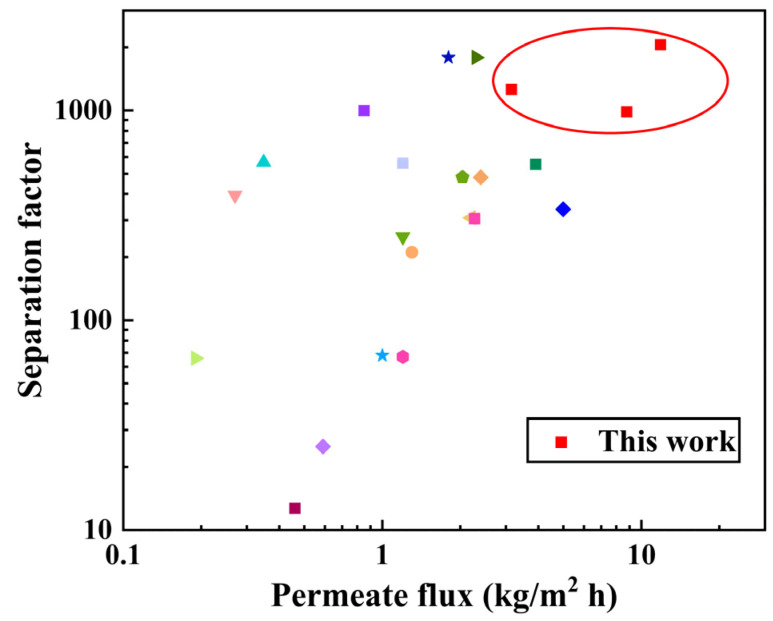
Summary of the performance of GO-based membranes for water/ethanol separation. Different colors and shapes represent the performance of reported GO-based membranes.

The anti-swelling property of as-prepared the PEI/GO/SC6 membrane was investigated at 343 K with the water content in feed increasing from 10 to 90 wt%. As shown in Figure 11, the permeate flux sharply increases from 3.13 to 11.84 kg/(m^2^·h) in the initial stage (water content from 10 to 30 wt%), while it almost remains constant in the range of 30–60 wt%. When the water content is higher than 60 wt%, the permeate flux shows an opposite trend with increasing water content: it remarkably declines from 13.47 to 6.77 kg/(m^2^·h). In general, the permeate flux significantly increases with increasing water content due to the improved driving force for water permeation and the decreased diffusion resistance arising from membrane swelling. However, a high water content in the membrane can facilitate the configurational rearrangement of polymeric chains (i.e., the relaxation of polymer chains), which may compact the membrane structure and lead to a decreased permeate flux. The similar variation in permeate flux under a high water content has also been observed in other polymeric membranes [28,29]. Herein, the declined permeate flux after the water content reaches 60 wt% indicates that the polymer relaxation plays a key role and the swelling of the membrane structure is mild. In the entire investigated range, the water content in the permeate fluctuates between 99.27 and 99.89 wt%. Especially when the water content in the feed is 30 wt%, the PEI/GO/SC6 membrane shows an excellent separation performance with a permeate flux of 11.84 kg/(m^2^·h) and a water content in the permeate of 99.89 wt%. It can be concluded that the PEI/GO/SC6 membrane shows excellent swelling resistance due to the strong electrostatic interactions. In addition, we summarized the water/ethanol separation performance of reported GO-based membranes (Figure 12). Comparatively, the PEI/GO/SC6 membrane shows an attractive performance due to the existence of SC6 molecules as water transport channels.

## 4. Conclusions

In this work, we achieved the incorporation of SC6 molecules into GO interlayer channels to promote the selective permeation of water molecules in water/alcohol separation process. Positively charged polyelectrolyte PEI was employed as a binder between GO and SC6 during a layer-by-layer assembly through forming electrostatic interactions, which confers a high SC6 content in the membrane and a stable membrane structure with excellent swelling resistance. The existence of SC6 molecules alleviates the tortuosity of mass transfer channels and then decreases the diffusion resistance. In addition, the unique structure of SC6 molecules with a hydrophilic entrance and a hydrophobic wall enables them to be ideal water transport channels, wherein the hydrophilic entrance provides high water affinity and ethanol repulsion, and the hydrophobic wall confers low resistance for water diffusion. The permeate flux and separation factor of the membrane are increased by 72% and 50%, respectively, after incorporating the SC6 molecules (with the permeate flux of 3.15 kg/(m^2^·h) and separation factor of 1260). The as-prepared membrane holds great potential in the application of solvent dehydration.

## Figures and Tables

**Table 1:** Effect of the substrate on the separation performance of PEI/GO/SC6 membranes.

Substrate	Permeate Flux (kg/(m^2^·h))	Separation Factor
PAN	2.01	265
hPAN	3.15	1260

**Table 2:** Permeance and selectivity of PEI/GO/SC6 membrane under different operation temperatures.

Temperature (K)	(P/l)_W_ (GPU)	(P/l)_E_ (GPU)	Selectivity
313	18,246	20.2	903
323	15,172	10.6	1431
333	11,814	9.9	1193
343	10,124	7.0	1446

## Data Availability

The raw data supporting the conclusions of this article will be made available by the authors on request.
